# Multi-Scale Squeeze U-SegNet with Multi Global Attention for Brain MRI Segmentation

**DOI:** 10.3390/s21103363

**Published:** 2021-05-12

**Authors:** Chaitra Dayananda, Jae-Young Choi, Bumshik Lee

**Affiliations:** 1 Department of Information and Communications Engineering, Chosun University, Gwangju 61452, Korea; chaitrad@chosun.kr; 2 Division of Computer Engineering, Hankuk University of Foreign Studies, Yongin 17035, Korea; jychoi@hufs.ac.kr

**Keywords:** CNN, tissue segmentation, multi global attention, brain MRI

## Abstract

In this paper, we propose a multi-scale feature extraction with novel attention-based convolutional learning using the U-SegNet architecture to achieve segmentation of brain tissue from a magnetic resonance image (MRI). Although convolutional neural networks (CNNs) show enormous growth in medical image segmentation, there are some drawbacks with the conventional CNN models. In particular, the conventional use of encoder-decoder approaches leads to the extraction of similar low-level features multiple times, causing redundant use of information. Moreover, due to inefficient modeling of long-range dependencies, each semantic class is likely to be associated with non-accurate discriminative feature representations, resulting in low accuracy of segmentation. The proposed global attention module refines the feature extraction and improves the representational power of the convolutional neural network. Moreover, the attention-based multi-scale fusion strategy can integrate local features with their corresponding global dependencies. The integration of fire modules in both the encoder and decoder paths can significantly reduce the computational complexity owing to fewer model parameters. The proposed method was evaluated on publicly accessible datasets for brain tissue segmentation. The experimental results show that our proposed model achieves segmentation accuracies of 94.81% for cerebrospinal fluid (CSF), 95.54% for gray matter (GM), and 96.33% for white matter (WM) with a noticeably reduced number of learnable parameters. Our study shows better segmentation performance, improving the prediction accuracy by 2.5% in terms of dice similarity index while achieving a 4.5 times reduction in the number of learnable parameters compared to previously developed U-SegNet based segmentation approaches. This demonstrates that the proposed approach can achieve reliable and precise automatic segmentation of brain MRI images.

## 1. Introduction

The segmentation of medical images plays a vital role in the study and treatment of many diseases. In particular, magnetic resonance imaging (MRI) is typically favored for structural analysis as it generates images with high soft-tissue contrast and higher spatial resolution and does not entail any health hazards. Brain MRI scans are quantitatively examined to diagnose various brain disorders such as epilepsy, schizophrenia, Alzheimer’s disease, and other degenerative disorders [1]. MRI is also essential to identify and localize abnormal tissues and healthy structures for diagnosis and postoperative analysis. Recently, convolutional neural networks (CNNs) have achieved exceptional performance in the field of computer vision, gaining widespread popularity owing to their ability to extract robust, non-linear feature representations. Deep network models have delivered exceptional performance in a broad spectrum of applications, including brain or cardiac imaging [2]. The typical architectures for brain MRI segmentation are fully convolutional neural networks (FCNNs) [3] or encoder-decoder-based networks [4,5]. These architectures typically include a contracting path, which represents an input image in terms of high-level feature maps, and an expanding path, where the feature maps are up-sampled in single or multiple up-sampling levels, to reconstruct a pixel-wise segmentation map [3,4,5]. Although they have powerful representation capabilities, these approaches tend to use redundant data flow, i.e., the networks extract identical low-level features multiple times at various network layers. In addition, the discriminative ability to represent features for pixel-wise labeling would be insufficient for challenging applications such as in medical image segmentation. To overcome these challenges and improve network accuracy, various schemes such as attention mechanisms, dilated convolutions, and multi-scale strategies have recently been proposed.

The attention mechanism allows the system to concentrate on the most salient features without additional monitoring. This limits the use of multiple identical feature maps and highlights the prominent features that are beneficial for a given task. It has been reported that attention modules benefit segmentation methods by creating an enhanced network for pixel-wise labeling [6,7,8,9]. Chen et al. [6] introduced an attention-based approach to influence multi-scale features acquired at different scales for the segmentation of natural images and showed increased segmentation performance over conventional methods for predicting multi-scale features. Despite the integration of attention modules in natural image segmentation, their application to medical images is restricted to simple attention models [10,11]. In addition to accuracy, many embedded applications consider the model size, energy consumption, and inference time to be significant in real-time use. A small CNN network enables on-chip model storage, resulting in less energy consumption during the recovery of parameters from dynamic random access memory during model training. Smaller CNNs also reduce the energy requirements for computation [12]. On the other hand, the off-chip memory contributes to the delay of hundreds of cycles of computation and dissipates more than 100 times as much energy as arithmetic operations. The reduction in memory and energy consumption allows the deployment of CNN models on less resource-intensive devices while retaining network precision.

To achieve higher segmentation accuracy with lower computation complexity, we propose deeper attention mechanisms, which are combined with multi-scale feature fusion that can enhance the performance of CNNs for the segmentation of brain MRI images. In particular, we propose a novel global attention module (GAM) at both the encoder and decoder sides with a multi-scale guided input. The multi-scale data extracted using 1 × 1 and 3 × 3 convolutional kernels jointly encode the complementary low-level information and high-level semantics from the extracted input patches. The feature maps from a previous network layer are provided together as input for the GAM on the encoder side with this multi-scale information. This proposed multi-scale strategy, along with the attention module, helps the encoder extract different semantic information. The GAM at the decoder helps capture discriminative information and focuses on relevant features while performing up-sampling operations. As a result, each network-layer contains two independent attention modules, which focus on extracting enhanced feature representations and generating an accurate segmentation network. The convolution blocks at the encoder and decoder layers are replaced with fire modules [13] that reduce the total number of model parameters.

Our main contributions are summarized as follows:

Contributions:

- We propose a modified U-SegNet architecture integrated with a novel global attention module. Attention is applied at both contracting and expansive paths, creating a multi-attention network. The key element in GAM is global average pooling, which provides the global context of high-level features as assistance to low-level features to obtain class-category localization.
- We propose a multi-scale input feature fusion strategy to extract the context information of high-level features at different scales, thus incorporating neighbor-scale feature information more precisely.
- The fire modules are used to replace the convolution layer to significantly reduce the number of model parameters, which results in a reduction in the model size and computation complexity; this consequently leads to a more efficient segmentation model.

The remainder of this paper proceeds as follows. Section 2 presents related work. Section 3 explains the proposed method and its architecture in detail. The experimental conditions, comparison studies, and comprehensive analyses are presented in Section 4. Finally, Section 5 concludes the paper.

## 2. Related Works

Extensive research has been conducted on medical image segmentation in the past ref. [13,14,15], with CNNs growing rapidly in this area, driving exceptional performances in many diverse applications. Conventional CNN architectures, including FCNN [3] or U-net [4], serve as sources of inspiration for existing medical image segmentation methods. The conventional FCNN-based classification network replaces the fully connected layers with convolutional layers to predict the output dense pixels. The input image is recovered to its original resolution by up-sampling the predictions in a single step. In addition, skip connections [3] are used in the network using intermediate function maps to boost the prediction capabilities. On the other hand, the U-net architecture consists of encoding and decoding paths with a sequence of convolutional layers with pooling and up-sampling. The features from the encoder are concatenated with the decoder layers using skip connections. Several extended U-net and FCNN models have been developed to resolve the problems associated with pixel-wise segmentation across different applications [16,17,18,19]. In [17], a patch-wise 3D U-net was proposed for brain tissue segmentation with encoding and decoding layers with randomly sampled and overlapped 3D patches (8 × 24 × 24) used for training. Unlike the U-net, a convolution operation is introduced as a transition layer between the encoder and decoder layers to give more weight to the higher-level features learned through deeper layers in the network. Pawel et al. [18] proposed a 3D-CNN for brain tumor segmentation, where the model was trained on 3D random patches, and features extracted by 2D-CNNs were given as an extra input to a 3D-CNN. The combination of both 3D and 2D features captures rich feature representations from a long-range 2D context in three orthogonal directions. An ensemble of 3D U-nets designed with different hyperparameters uses non-uniformly extracted patches as inputs to obtain brain tumor segmentation [18]. Badrinarayanan et al. [20] introduced the SegNet model, which uses pooling indices from the encoder to the up-sampling layers. Hence, it requires very few parameters and is faster to train. Looking into the complementary strengths in both.

SegNet and U-net models, a new hybrid model is explored, namely U-SegNet [21]. The U-SegNet incorporates the unique architectural features from both U-net and SegNet models and uses SegNet as the base architecture with a skip connection introduced between the encoder and decoder, providing multi-scale information for better performance. Owing to the pooling indices passed at the decoder side, the U-SegNet model has faster convergence. Recent efforts to promote the discriminative capability of feature representations include a multi-scale fusion strategy [22]. Zhou et al. [23] redesigned the skip connections in U-net++ [23] by enabling flexible feature fusion in decoders, thus resulting in an improvement over the restrictive skip connections in U-net [4] that require fusion of only same-scale feature maps. A small drawback in U-net++ is that the number of parameters increases owing to the employment of dense connections [24,25]. Deep supervision is also employed to balance the decline in segmentation accuracy caused by pruning [26]. Zhao et al. [27] proposed a pyramid network that utilizes global learning at various scales by grouping feature maps produced by multiple dilated convolution blocks. The collection of contextual multi-scale information can also be obtained by performing pooling operations [28]. Cheng et al. [29] proposed a context encoder network (CE-net) that adopts a pre-trained ResNet block in the feature encoder. CE-net involves a newly proposed dense atrous convolution block and residual multi-kernel pooling is integrated into the ResNet-modified U-net structure to capture more high-level features and preserve more spatial information. Although these approaches assist in capturing targets at different measures, contextual dependencies for all image regions are uniform and non-adaptive. Hence, these approaches neglect the contrast between local and contextual representations for different categories. Moreover, these multi-context representations were manually composed and lacked the flexibility to form multi-context representations. This causes long-range object associations in the entire image to be leveraged in these strategies, which is of focal interest in many medical image segmentation problems.

The attention mechanisms highlight key local regions in the feature maps and discard unrelated data carried by the generated feature maps. The attention modules act as crucial components of a network that wants to gather global information. The inclusion of the attention blocks demonstrated very successful outcomes in various vision problems, such as image classification [30], image captioning [31], or image question-answering [32]. Recently, many researchers have shown interest in self-attention, as they offer a greater opportunity to model long-range dependencies while retaining computational and statistical performance [33,34,35,36]. Zhao et al. introduced a point-wise spatial attention network, where each position on the feature map is connected to all the other feature maps through a self-adaptively learned attention mask [37]. Dong et al. [38] proposed attention gates (AGs) and used them for the segmentation of the pancreas. The AGs highlight the salient features while suppressing the irrelevant region from the raw input pixel. AGs utilize intermediate features more effectively, thus minimizing the use of cascaded models [39]. Wang et al. [40] used a simple focus module with three convolutional layers to combine local and global-dependent features. A similar focus module with two convolutional layers integrated with a U-net architecture was proposed in [41]. For better extraction of relevant features, focus gate modules are integrated with the skip connection in the decoding path of the U-net in [39]. Schlemper et al. [41] proposed attention modules where the local deep attention features are fused with the global context at multiple resolutions for prostate segmentation on ultrasound images. The multi-scale self-guided attention-based approach can integrate local features with their respective global dependencies, as well as highlight interdependent channel maps in an adaptive manner to achieve accurate segmentation of medical images [42].

Most of the deep learning-related studies have considered increasing network accuracy as their main objective. However, the computational burden of a significant number of parameters and deep architecture becomes a crucial issue. Recent studies have shown that most deep neural networks are over-parametrized, resulting in deep learning network redundancy, which leads to inefficient use of memory and computing resources. In these large parameter spaces, various compression techniques, such as shrinking, factorizing, or compressing pre-trained networks, are applied to minimize redundancy and obtain smaller models [43,44,45,46]. In [44], singular value decomposition (SVD) was used for a pre-trained CNN architecture to achieve lower-order parameter estimates for model compression. Network pruning methods [43,45] have been widely studied to achieve compressed CNN models. The parameters of the pre-trained model below a certain threshold are replaced with zeros in the network pruning method to produce sparse matrices. Most of the previous works [45,46] introduced network-pruning-based methods to decrease the network complexity and reduce the overfitting of the model. Network quantization is proposed to decrease the data dynamic range from 32 to 8 or 16 bits, which further compresses the pruned network by reducing the number of bits required to represent each weight [47]. To efficiently operate on compressed deep learning models, Son et al. [48] proposed an efficient inference engine (EIE), a specialized accelerator that accomplishes customized sparse matrix-vector multiplication and performs weight sharing without efficiency loss. To reduce the CNN parameters and computational work, various methods based on factorizing the convolution kernel have been introduced [49]. The depth-wise separable convolutions used in SqueezeNet [13,50] are a form of factorizing convolution that separates convolution across channels rather than convolution within channels. As in the MobileNetV1 architecture, the profoundly separable convolution networks realized with quantization require a special attention module [51]. The special hardware for CNNs has been considered by many methods aimed at minimizing computation time [50].

## 3. Proposed Method

It is known that the SegNet [20] and U-net [4] are the most widely used deep learning models for image segmentation [21,25]. In SegNet, pooling indices in the up-sampling process are utilized to compensate for the missing spatial information and lead to faster convergence of the model [20]. U-net uses skip connections from the encoder to the decoder and shows a better segmentation performance [4]. However, multi-stage cascaded CNN approaches are more suitable because the performance of a single SegNet and U-net-based segmentation method is not sufficiently accurate when there are variations in the structure and intensity of the target tissues [25]. However, multiple cascaded networks can produce a significantly large number of model parameters, thus leading to the redundant use of computational resources. To overcome this problem, U-SegNet [21] uses both skip connections and pooling indices to combine feature maps from the encoder to the decoder and localize these feature up-sampling, respectively. As a result, pooling indices make the U-SegNet converge faster and the skip connection improves the segmentation accuracy.

Although U-SegNet shows better segmentation performance, the segmented outputs are still blurry, and the network is insensitive to the fine details of the image [24]. To achieve a better segmentation of brain tissues when training on a limited set, the network needs to extract more discriminative features [38]. However, U-SegNet is slightly insufficient to capture better features because numerous pooling operations in the U-SegNet model produce low-resolution feature maps. Due to the inherent complexity, a large number of layers, and the massive amounts of data required, deep learning models are very slow to train and require a lot of computational power, which makes them very time- and resource-intensive. The model which can provide improved segmentation results while training on limited or less data is considered to be a highly potential network [52]. Meanwhile, due to data scarcity, the need to develop a model which could be trained efficiently on less data is very crucial [53]. Motivated by this problem, we propose a novel global attention mechanism using a U-SegNet architecture, where the proposed architecture is designed with a multi-scale guided multi-global attention module. The multi-scale input features at each encoding layer encode both the global and local contexts. Moreover, the proposed novel global attention at the encoder and decoder can filter irrelevant information and focus on the most relevant details needed for the MRI segmentation task. Besides, the model is prone to lose local details when complete image information is employed as an input to train the network. We also propose the use of a patch-wise splitting of each input slice to resolve this problem, which is used to train the model and provide better segmentation accuracy. Finally, we adopt fire modules that comprise a squeeze layer consisting of only 1 × 1 convolution filters followed by an expansion layer with a combination of 1 × 1 and 3 × 3 filters, which reduces the number of learnable parameters and computational requirements, and results in a smaller efficient model.

**Figure 1:**
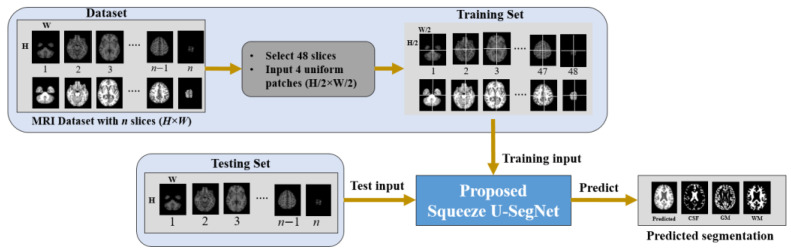
Overall framework of the proposed algorithm.

**Figure 2:**
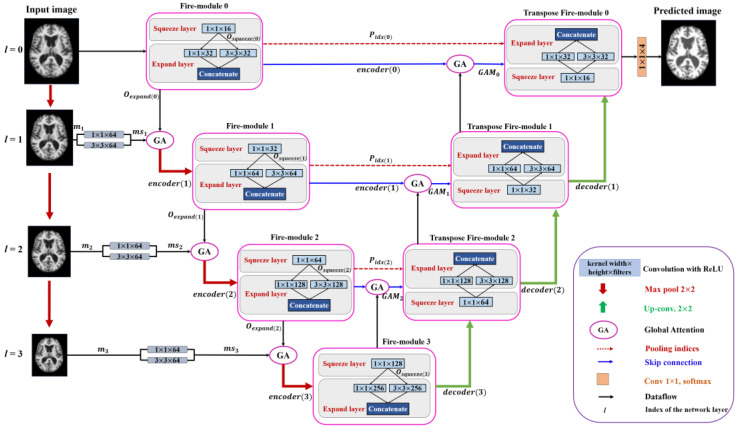
Overview of our multi-scale squeeze U-SegNet with multi global attention for brain MRI segmentation. Solid blue boxes represent the convolution block with dimensions as kernel width × height × filters followed by ReLU activations.

Figure 1 shows the overall framework of the proposed method. First, the MRI datasets with the corresponding ground truth are prepared. For each MRI scan with the dimension of height × width × slices (*H* × *W* × *S*)*,* we pad zeros to the *H* × *W* of each slice and resize to a dimension of 256 × 256. Then, 48 slices are extracted starting from the 10-th slice with an interval of 3 slices [54]. Furthermore, each slice is divided into four uniform non-overlapping patches and these patches are given as input to the proposed model for training. Figure 2 shows the end-to-end encoder-decoder architecture of the proposed method. As shown in Figure 2, the features extracted using 1 × 1 and 3 × 3 filters are fused to form a multi-scale input representation. These multi-scale data with the feature maps from a previous network layer are provided as input to the GAM at the encoder side. The GAM at the decoder can capture discriminative information and concentrate on relevant features while performing up-sampling operations. Thus, each network-layer contains two separate attention modules which concentrate on extracting enhanced representations of features and generate an accurate segmentation network. In addition, the convolution blocks at the encoder and decoder layers are replaced with fire modules which reduce the number of model parameters and create a smaller network. The architecture of the proposed method is discussed in detail as follows: (i) encoder path, (ii) decoder path, and (iii) global attention module (GAM), (iv) fire module, (v) uniform patch-wise input, and (vi) classification layer.

### 3.1. Encoder Path

Figure 2 shows the architecture of the proposed method with the encoder and decoder paths. The fire module replaces the convolution operation in the proposed method, significantly reducing the number of learnable parameters and computational complexity. The fire module was originally used for SqueezeNet [13] to reduce the complexity of AlexNet [50]. In this study, we incorporate it with our proposed multi-scale U-SegNet architecture for segmentation. Let us consider $x_{l}$ as the input sample, where *l* represents the index of the network layer. The convolution output for the squeeze block is computed as (1).

(1) $$o_{squeeze\left(l\right)}=f\left(x_{l}*w_{l}^{1\times 1}+b_{l}\right)$$

where $o_{squeeze\left(l\right)}$ is the squeeze layer output of the fire module and $w_{l}^{1\times 1}$ is the kernel weight, where the subscript [1 × 1] represents the size of the convolution kernel associated with the respective layer and $b_{l}$ is used as a bias term. * represents the convolution operation. The convolution output is fed to the standard Rectified Linear Unit (ReLU) activation function *f* (∙).

The squeeze output is fed into the expanding module. The expanding module consists of two parallel convolutions with kernel sizes of 3 × 3 and 1 × 1. Furthermore, the output from these parallel convolutions is concatenated to form the fire module output, and is expressed as (2).

(2) $$o_{expand\left(l\right)}=\mathrm{Concat}\;[f\left(o_{squeeze\left(l\right)}*w_{l}^{1\times 1}+b_{l}\right),\;f(o_{squeeze\left(l\right)}*w_{l}^{3\times 3}+b_{l})]$$

where $o_{expand\left(l\right)}$ is the fire module output of the l-th network layer, and Concat() is a concatenate function. As shown in Figure 2, the encoder path consists of a sequence of fire modules whose output is applied to the GAM as input. The GAM also receives input $ms_{l}$, obtained from multi-scale input feature fusion. In the multi-scale layer, the input $x_{l}$ is down-sampled using max-pooling with a stride of 2 × 2, as in (3).

(3) $$m_{l}=Maxpool(x_{l-1},\;2)$$

The max-pooled input is followed by a convolution of 1 × 1 and 3 × 3 filters separately. These convolved outputs are concatenated to form multi-scale feature maps, as shown in (4). The multi-scale information and the fire module output and are fed as input to the GAM at each encoding layer, as in (5).

(4) $$ms_{l}=Concat[f(m_{l},\;w_{l}^{1\times 1}),\;f(m_{l},\;w_{l}^{3\times 3})],$$

(5) $$GAM_{l}=GAM(ms_{l},\;o_{expand\left(l\right)}),$$

The output from GAM is given to the max-pooling layer to reduce the dimensionality and focus on the fine details of the feature map, as expressed in (6). Pooling indices are stored at each encoder layer so that the decoder uses the information to up-sample the feature maps. The output at each encoder layer is referred to as the encoding unit (down-sampling unit) and is obtained using (6).

(6) $$encoder\left(l\right)=Maxpool(GAM_{l},\;2),$$

### 3.2. Decoder Path

Similar to the encoding path, the decoder path in the proposed method uses transposed fire modules to reduce the number of model parameters. The main component of the decoder path is the up-sampling unit. Each up-sampling unit consists of a transposed fire module. The transposed fire module consists of a 1 × 1 transposed convolution. The output from the 1 × 1 transposed convolution is fed into two 3 × 3 and 1 × 1 kernel-sized parallel transposed convolutions that are concatenated to form the output transpose fire module, as in the down-sampling unit. The decoder is integrated with attention gates, which can highlight the salient features. The feature maps extracted from the l-th (high-level) and (l−1)-th (low-level) encoding layers are used as the input signal and gating signal to the attention module, respectively. Thus, the feature map obtained from encoding layers containing contextual information is computed using the GAM to eliminate unrelated responses. The GAM output is concatenated with the feature map of the corresponding up-sampling layer, as expressed in (7). Hence, the attention-based skip connections in the proposed architecture help in extracting the most informative data from the encoder, utilized by the decoder to make more accurate predictions. These skip connections use both high-and low-resolution feature information and focus on the most relevant information while performing up-pooling operations.

(7) $$decoder\;\left(l\right)=Concat\left[GAM(x_{l},x_{l-1}\right),\;Unpool\;\left(x_{l-1},Pool_{idx\left(l-1\right)}\right)]$$

The output from each decoder layer can be obtained using (7), where $Pool_{idx\left(l-1\right)}$ is the pooling index passed from the encoder layer to the decoder layer to recover spatial information of the feature maps while performing un-pooling operations at the decoder.

### 3.3. Global Attention Module (GAM)

A distinctive brain signal processing system for human vision is the visual attention process. This is a tool for a human to pick relevant information instantly from a large amount of information using sources of limited attention. In deep learning, the attention process is similar to that of human visual attention. Its main objective is to determine the most relevant data from a vast amount of knowledge to accomplish its goal (tissue segmentation). By suppressing function activations that are not important to the task, the attention mechanism improves the network performance. To do this, we propose a novel global attention module with self-attention in an efficient manner. As a guide from low-level features to assess class localization, the GAM on each encoder and decoder layer enables global context details.

**Figure 3:**
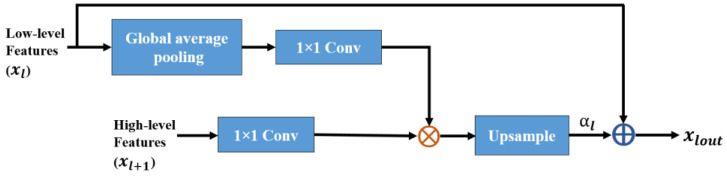
Schematic of the proposed global attention module (GAM) integrated into the proposed brain segmentation architecture.

Figure 3 shows the proposed architecture of GAM, which is integrated with our proposed brain segmentation architecture. The $x_{l}$ is the output feature map from the l-th encoding layer (low-level features). The $x_{l+1}$ collected from a coarser scale serves as a vector of the gating signal and is applied to select the target area for each pixel. The $\alpha_{l}$ is the tensor coefficient that preserves activation by suppressing irrelevant feature responses associated with the target task. The operation of GAM is the element-wise addition of the feature map with the tensor coefficient from the l-th encoding layer and the output of GAM is obtained using (8).

(8) $$x_{lout}=\alpha_{l}+x_{l}$$

In the case of multiple semantic groups, learning multidimensional coefficients of attention is suggested. As guidance for low-level features to incorporate local features into the global context, global average pooling provides global context information. The global information produced from the high-level feature is fed to a 1 × 1 convolution with the ReLU activation function. To obtain weighted low-level characteristics, it is multiplied by 1 × 1 convoluted low-level features. To obtain the tensor coefficient of attention, we used multiplicative attention. To extract pixel localization specific to the class of the high-level feature index, the tensor coefficient is up-sampled and added with low-level features. The tensor coefficient of attention is obtained as follows:

(9) $$\alpha_{l}=upsample\left\{\left(GAP\left[x_{l}\right]*W_{x}+b\right),\;\left(x_{l+1}*W_{g}+b\right)\right\},$$

where $W_{x}\;\mathrm{and}\;W_{g}$ are the weight values associated with the input and gating signals, respectively, *b* is the bias term, and *GAP* is a function of the global average pooling. The feature maps obtained from the attention module $x_{lout}$, which contains contextual information, were concatenated with the feature map of the corresponding decoding layer forming skip connections. These skip connections use both high- and low-resolution features; they focus on the most relevant information while performing up-sampling operations.

### 3.4. Fire Module

**Figure 4:**
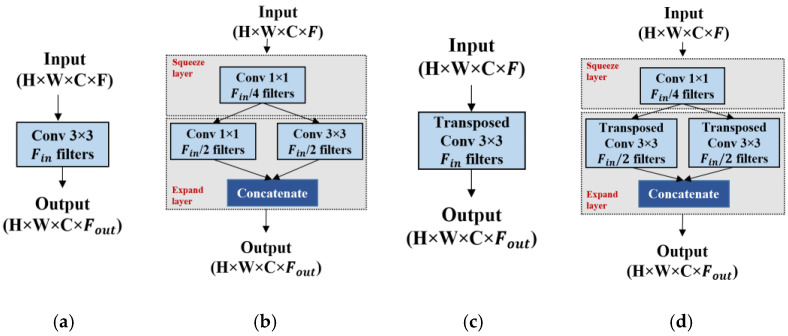
Schematic of fire module. (**a**,**c**) show the convolution for the encoder and decoder side in U-net [4] respectively; (**b**,**d**) show our corresponding squeeze U-SegNet at encoder and decoder side using squeeze and expand layers to reduce the number of parameters.

The fire module was initially introduced in SqueezeNet [13] to identify CNN architectures with fewer parameters while maintaining competitive accuracy. The fire module in SqueezeNet is composed of a squeeze convolution layer with only 1 × 1 filters, feeding into an expand layer with both 1 × 1 and 3 × 3 convolution filters. The number of 1 × 1 filters in the squeeze layer is set to be less than the total number of 3 × 3 and 1 × 1 filters in the expand layer, so the squeeze layer helps in limiting the number of input channels to the 3 × 3 filters in the expand layers. Owing to the benefits of the fire module in reducing the learnable parameters, we used the same design of the fire module and integrated it into our proposed encoder and decoder architecture. Figure 4 shows a schematic representation of the fire module applied to the proposed architecture. Figure 4a,c show the encoder and decoder sides of U-net [4] using a normal convolution layer, with each convolution block containing $F_{in}$ filters, and takes a feature map of height × width × channels (H × W × C) as input. Figure 4b,d shows the architecture of the fire modules at the encoder and decoder paths in the proposed method. Likewise, the fire module of SqueezeNet, the fire module in the proposed method consists of two parts: (i) the squeeze layer and (ii) the expand layer. As shown in Figure 4b, the squeeze module consists of one convolution layer with a kernel size of 1 × 1 and an output channel equal to $F_{in}/4$, where $F_{in}$ is the number of convolution filters in the conventional U-net [4]. The squeeze output is fed into the expanding module. The expanding module consists of two parallel convolutions with kernel sizes of 3 × 3 and 1 × 1, each convolution with $F_{in}$/2 output channels. Furthermore, the output from these parallel convolutions is concatenated to form a fire module output, where $F_{out}=F_{in}$. Hence, as mentioned in [13], the proposed method maintains the number of filters in the squeeze layer, which is less than the total number of filters in the expand layers, which results in a significant reduction in the total number of network parameters.

### 3.5. Uniform Patch-Wise Input

The brain MRI scan of each subject constitutes the dimensions H × W × S. Some of the starting and ending slices of the brain MRI scan do not provide much useful information, as analyzed in the previous research [54], and the consecutive slices would share almost the same information. Hence, to exclude these non-informative slices and reduce the multiple training of consecutive slices, we selected 48 slices with a gap of 3 slices, which ensured the presence of slices with more as well as less information for model training. Each of the extracted slices was resized to 256 × 256. The partitioning of a slice with individual patches improves localization because the trained network can better concentrate on local details in a patch. Therefore, each slice was divided into four uniform patches using our proposed method. Therefore, the dimensions of each partitioned patch were 128 × 128 in the proposed method. These patches are fed into the training of the model and the predicted segmentation results are obtained for the test data.

### 3.6. Classification Layer

The final decoder layer consists of a 1 × 1 convolutional layer with softmax activation to predict a reconstructed segmentation map. The output contains four predicted classes: gray matter (GM), white matter (WM), cerebrospinal fluid (CSF), and background. The proposed model accepts the input image and produces the corresponding learned representation. Based on this feature representation, the input image is classified into any of the four output classes. The cross-entropy loss is used to measure the proposed model losses, as in (11). The softmax layer learns representations from the decoder and interprets them in the output class. The probability score $y^{\prime }$ is assigned to the output class. If we define the number of output classes as c, we obtain as (10) as follows:

(10) $$y^{\prime }=\frac{exp^{decoder\left(l\right)}}{\sum_{j=1}^{c}exp^{decoder{\left(l\right)}_{j}}}\;$$

and the cross-entropy loss function is used to calculate the network cost function as in (11):

(11) $$L(y,y^{\prime })=\sum_{i=1}^{c}y_{i}log(y_{i}^{\prime })$$

where for each class of *i*, the ground truth and predicted distribution score are *y* and $y^{\prime }$, respectively.

## 4. Experimental Results

The proposed method was evaluated using two sets of brain MRI images. The first sample included 416 T1 weighted brain MRI scans from the Open Access Series of Imaging Studies (OASIS) database [55], where information from both non-demented and demented subjects was obtained from Washington University. A T1-weighted (T1W) image is a basic pulse sequence in magnetic resonance (MR) imaging and depicts differences in signal based upon intrinsic T1 relaxation time of various tissues. Clinically, T1-weighted images generally are better for depicting normal anatomy and are mainly used for the anatomical details and pathological abnormalities of the intracranial lesions [56]. Of the 416 subjects in total, 150 were chosen for our experiments. The first 120 subjects were used for model training out of the selected data and the remaining 30 subjects were used as test datasets. For our studies, the axial, sagittal, and coronal planes of the MRI slices were used for training and testing the proposed network. In the OASIS dataset, the size of each input axial scan was 208 × 176 × 176 which corresponds to height × width × slices respectively and each scan consisted of 176 slices. It was observed that the distinguishable tissue regions were mostly found near the middle slices of the volume [54]. Often, the same information is exchanged for consecutive slices. Therefore, to remove these non-informative slices and decrease the repetitive training of consecutive slices, a sample of 48 slices, starting from the 10-th slice with an interval of three slices, were selected for the evaluation procedure. By inserting 24 pixels of zeros at the top and bottom of the image and 40 pixels of zeros on the left and right of the image, the extracted slices were resized to the dimensions of 256 × 256 × 48. Similarly, the sagittal and coronal planes of MRI slices were also resized to 256 × 256 dimensions. Each input scan, therefore, consisted of 48 slices with dimensions of 256 × 256. During the training phase, slices of each MRI scan and their corresponding ground-truth segmentation maps were split into uniform patches. An input slice had dimensions of 256 × 256 and each slice was split into four patches. Therefore, in the proposed model, the dimensions of each partitioned patch were 128 × 128. These patches were given as input to the training model and the predicted segmentation results were obtained for the test data.

The second dataset contains MRI from the Internet Brain Segmentation Repository (IBSR) [57] dataset. The IBSR dataset includes 18 T1-weighted MRIs of 14 healthy men and 4 healthy women between 7 and 71 years of age. The MRIs in the IBSR are provided after pre-processing, such as skull stripping, normalization, and bias field correction. The training dataset included 12 subjects with manually annotated and confirmed ground truth labels for the remaining six subjects for testing the model. The original axial scans (256 × 128 × 256) were padded to the top and bottom of the image with 64 pixels of zeros to resize to a dimension of 256 × 256 × 256 to use the patches effectively in our proposed model. Similarly, the original coronal (256 × 256 × 128) and sagittal (128 × 256 × 256) scans were also resized to dimensions of 256 × 256 × 256 for the experiments. Table 1 summarizes the OASIS and IBSR datasets used in the experiments.

The training and testing were performed on an NVIDIA GeForce RTX 3090 GPU to build the proposed network and use stochastic gradient descent to optimize the loss function. For training, we set the learning rate to 0.001, a high momentum rate of 0.99, and the number of epochs to 10. The Keras framework for implementation of the proposed work was used.

**Figure 5:**
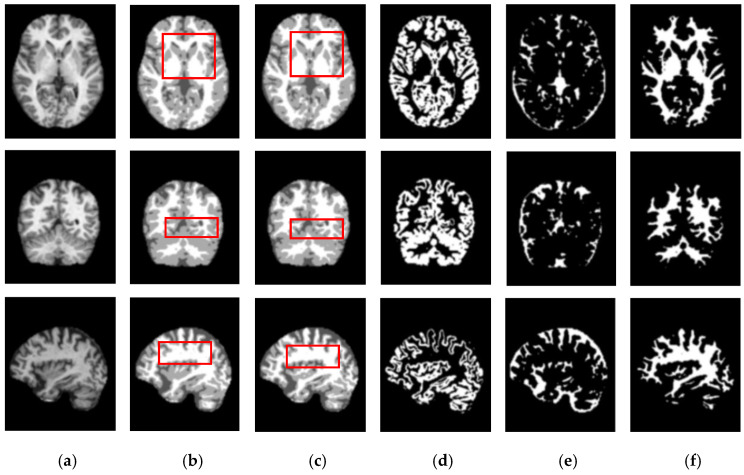
Segmentation results for the axial, coronal, and sagittal planes of the brain MRI image (top to bottom) on the OASIS dataset using the proposed method. (**a**) Original input images, (**b**) ground truth segmentation map, (**c**) their predicted segmentation map obtained by using the proposed method, (**d**) predicted GM (binary map), (**e**) predicted CSF (binary map), (**f**) predicted WM (binary map).

**Figure 6:**
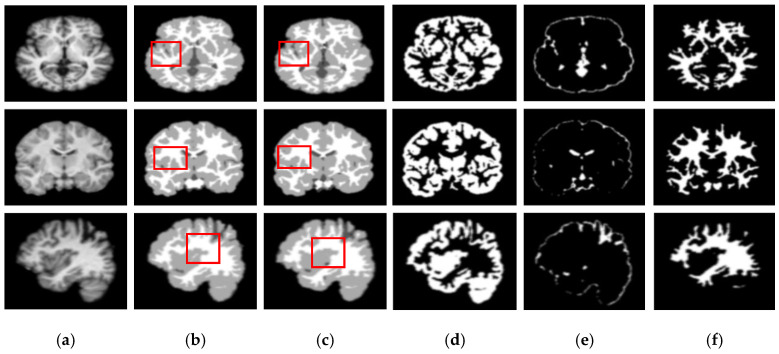
Segmentation results for the axial, coronal, and sagittal planes of the brain MRI image (top to bottom) on the IBSR dataset using the proposed method. (**a**) Original input images, (**b**) ground truth segmentation map, (**c**) their predicted segmentation map obtained by using the proposed method, (**d**) predicted GM (binary map), (**e**) predicted CSF (binary map), (**f**) predicted WM (binary map).

Figure 5 and Figure 6 show the segmentation results for the axial, coronal, and sagittal planes of the OASIS and IBSR datasets, respectively. The figures show that the proposed approach achieves well-segmented performances for GM, WM, and CSF of the brain MRI on both datasets. The axial plane shows the most informative details in the central slices of the MRI compared to the other planes. Thus, the segmentation results for the axial planes show the segmentation performance most effectively. In addition, the highlighted boxes in Figure 5 and Figure 6 show that the quality of sagittal and coronal images is highly promising without any difference in every detail. From the results of Figure 5 and Figure 6, it can be inferred that the proposed method can extract complicated pattern features from all three planes.

We evaluated the performance of the proposed method using quantitative metrics. Table 2 lists the DSC values [58] and JI [59], which are popular methods for comparing the ground truth and segmented results. DSC is defined as twice the number of elements common to both sets divided by the sum of the number of elements in each set, where |X| and |Y| are the cardinalities of the ground truth set and predicted segmentation set (i.e., the number of elements in each set). JI is expressed in terms of the DSC, as listed in Table 2. Both DSC and JI metrics determine the match between the predicted segmentation map and the corresponding ground-truth segmentation map.

**Figure 7:**
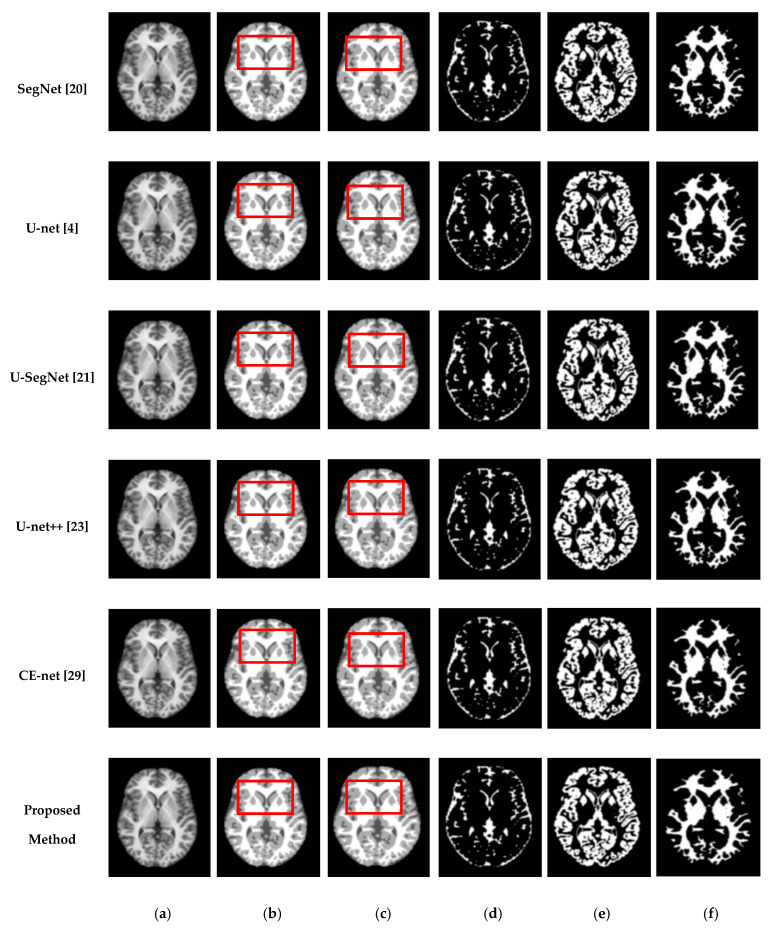
Segmentation results for GM, CSF, and WM from brain MRI image using the existing methods and the proposed method on OASIS dataset: (**a**) original input image; (**b**) ground-truth segmentation map; (**c**) their segmentation results obtained SegNet, U-net, U-SegNet, U-net++,CE-net, and the proposed method (**top** to **bottom**); (**d**) CSF maps obtained by SegNet, U-net, U-SegNet, U-net++,CE-net, and the proposed method (**top** to **bottom**); (**e**) GM maps obtained by SegNet, U-net, U-SegNet, U-net++,CE-net, and the proposed method (**top** to **bottom**); (**f**) WM maps obtained by SegNet, U-net, U-SegNet, U-net++,CE-net, and the proposed method (**top** to **bottom**).

**Figure 8:**
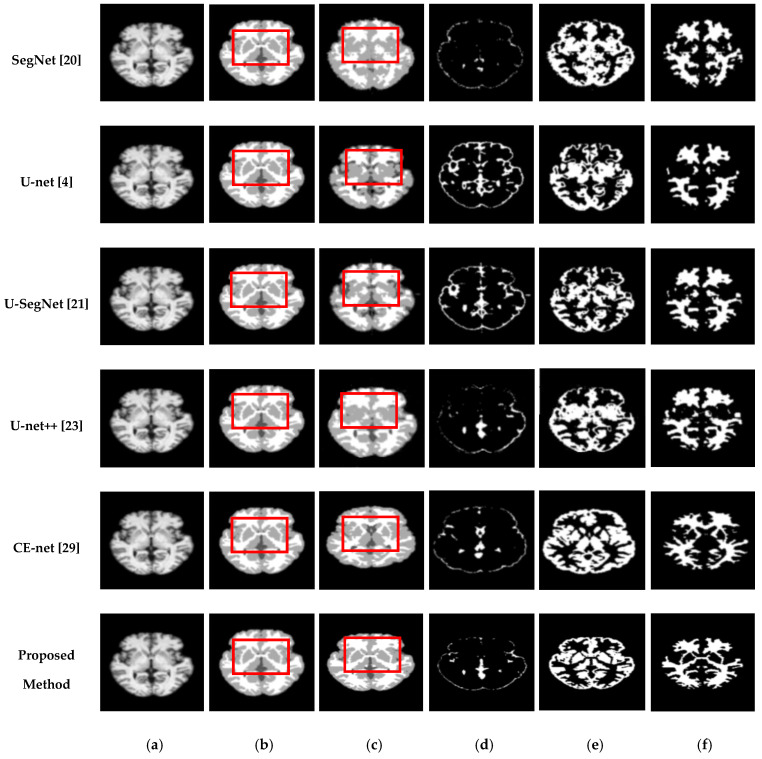
Segmentation results for GM, CSF, and WM from brain MRI image using the existing methods and the proposed method on IBSR dataset: (**a**) original input image; (**b**) ground-truth segmentation map; (**c**) their segmentation results obtained SegNet, U-net, U-SegNet, U-net++,CE-net, and the proposed method (**top** to **bottom**); (**d**) CSF maps obtained by SegNet, U-net, U-SegNet, U-net++,CE-net, and the proposed method (**top** to **bottom**); (**e**) GM maps obtained by SegNet, U-net, U-SegNet, U-net++,CE-net, and the proposed method (**top** to **bottom**); (**f**) WM maps obtained by SegNet, U-net, U-SegNet, U-net++,CE-net, and the proposed method (**top** to **bottom**).

We also assessed the segmentation performance in terms of the mean square error (MSE), which is the average square difference between the original and predicted Y values. The Hausdorff distance (HD) [60] was used to determine the dissimilarity between two sets in a metric space. The two sets of small Hausdorff distances are almost identical. HD and MSE are computed as listed in Table 2, where D is the Euclidean distance between two pixels, and R and C are the image height and width, respectively. To compare the segmentation results of various network architectures, we experimented on SegNet, U-net, U-SegNet, U-net++, and CE-net models under the same experimental conditions. Figure 7 and Figure 8 show comparisons of the segmentation results. As shown in Figure 7 and Figure 8, the proposed method shows superior results in terms of the quality of the segmentation map compared to those of other conventional methods. Although the skip connections in the U-net improve feature representations by combining low-level and high-level information, they suffer from a large semantic gap between low- and high-resolution feature maps, resulting in high misclassification rates of brain tissues. Furthermore, for medical images with low contrast, blurred boundaries between different tissues, the segmentation accuracies of U-net and SegNet are significantly degraded. Because the network layers in U-net++ are connected through a series of nested, dense skip pathways, leading to redundant learning of features, they did not show good performance. In particular, it can be observed that there are misclassification results in the feature maps generated by SegNet, U-net, and U-net++ in the red boxes of Figure 7c and Figure 8c. Although U-SegNet with pooling indices and skip connections yields better segmentation results, it fails to capture fine details, as shown in Figure 8c. From the highlighted red boxes in Figure 8, it can be observed that U-SegNet fails to identify differences between WM and GM tissues, and most of the GM tissues are incorrectly predicted as WM. The CE-net extracts multi-scale information through a context encoder block for the segmentation of medical images. However, the context encoder block is employed only at the bottleneck layer of the model, and thus this multi-scale information could be irrelevant by the time it reaches the final decoder layer for classification. To overcome these limitations, we extract multi-scale information at each network layer followed by the GAM to enhance the segmentation performance by directing attention to related areas. This improved segmentation can be observed in the results obtained using the proposed method. Similar results were observed for the segmentations obtained from the IBSR images, as shown in Figure 8. It can be observed that the proposed network obtains finer details than the other architectures. These results indicate that our proposed approach can strongly recover finer segmentation details while bypassing distractions between tissue boundary regions.

The quantitative analysis of the proposed method is performed in comparison with SegNet [20], U-net [4], U-SegNet [21], U-net++ [23], and CE-net [29]. Table 3 lists the comparative results in terms of the average and standard deviation of DSC, JI, and HD metrics, respectively As listed in Table 3, the proposed network achieves significant improvements of 10%, 3%, 2%, 2%, and 1% (in terms of DSC) over SegNet [20], U-net [4], U-SegNet [21], U-net++ [23], and CE-net [29], respectively, and obtained a lower MSE value of 0.003 on average. In addition, the maximum standard deviations for DSC, JI, and HD are 0.098, 0.096, and 0.088, respectively, which are close to the mean values; this indicates that the pixel predicted values are fitted well to the ground truth values without much data variation. For each encoder map, SegNet [20] stores only the max-pooling indices, i.e., the maximum feature value positions in each pooling window are stored and used for up-sampling. This improves boundary delineation with 3.5 million parameters with approximately 1.4 *h* of training time, requiring fewer resources among the existing methods in our proposed method. SegNet tends to miss several fine details because when performing up-sampling from low-resolution feature maps, it loses adjacent information. On the other hand, U-net uses skip connections as the core of the architecture, which blends deep, coarse information with shallow, fine semantic information. A drawback of U-net is its significant memory requirement because lower-level features in the up-sampling process must be stored for further concatenation. Because U-net uses low-level feature maps for up-sampling, translation invariance is often compromised. Moreover, U-SegNet [21] tends to be insensitive to fine details, and it is evident from the difficulty in identifying boundaries between adjacent tissues, such as WM and GM. The design of atrous convolution followed by multi-kernel max-pooling in the CE-net helps to capture multi-scale information and avoids the acquisition of redundant information. However, the multi-scale feature extraction capability of the CE-net is limited to the bottleneck layer, leading to poor feature presentation at the final decoder layer. The segmentation maps generated by these existing models have a relatively low resolution because of the pooling layers in the encoder stage. Hence, to preserve the high spatial resolution, the pooling layers must be removed. However, because convolution is a rather local operation, SegNet, U-net, U-SegNet, U-net++, and CE-net models would not be able to learn holistic features in the images without pooling layers.

Our proposed method presents a multi-scale feature fusion scheme combined with GAM as a potential solution to the problems discussed above and produces improved segmentation accuracy. The max-pooled output was filtered with 1 × 1, 3 × 3 kernels. Then, they are concatenated together and can extract the global context without losing the resolution of the segmentation map. In this way, global information can be exchanged between layers without reducing the resolution, leading to lowered blurring in the segmentation maps. In addition, the GAM at the encoder enables the presentation of global context information as a guide for low-level features to extract the original resolutions for segmentation. The GAM at the decoder shows that the combination of global features and local features is essential to discriminate brain tissues and is consistent with the results from previous studies. Furthermore, uniform input patches allow the network to concentrate better on local information. As a result of the selective integration of spatial information through uniform patches, feature maps followed by multi-scale guided multiple GAMs help in capturing context information and can efficiently encode complementary information to segment the brain MRI accurately.

**Figure 9:**
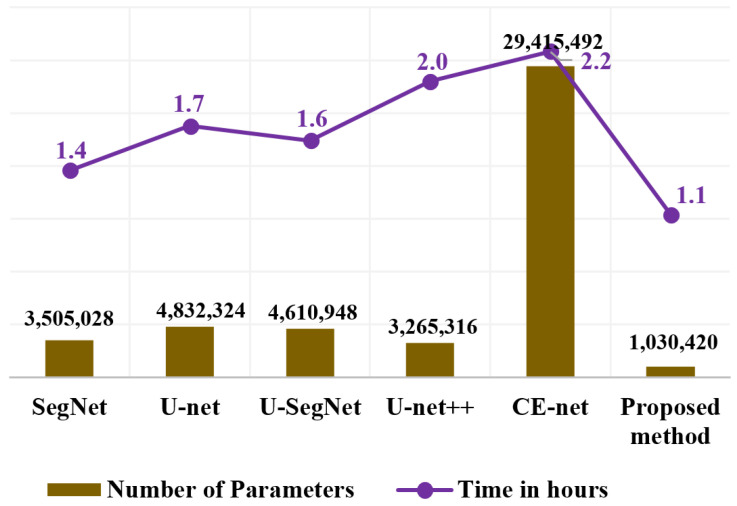
Detailed data on the number of learnable parameters and computation time for the proposed and conventional methods for the OASIS dataset.

As mentioned above, we propose the use of a fire module for fewer learnable parameters while maintaining equivalent accuracy. Figure 9 shows the details of the number of learnable parameters and computation time consumed by the proposed method in comparison with conventional methods. Smaller models can be built by arranging a sequence of fire modules that consist of a squeeze layer that has only 1 × 1 convolution filters. This serves as an expansion layer that has a combination of 1 × 1 and 3 × 3 filters. The number of filters in the squeeze layer was defined to be less than the number of 1 × 1 and 3 × 3 filters in the expand layer. The 1 × 1 filters in the squeeze layer down-sample the input channels and decrease the parameters before they are given as an input to the expand layer. The expansion layer consists of both 3 × 3 and 1 × 1 filters. The 1 × 1 filters in the expand layer combine channels and perform cross-channel pooling, but cannot recognize spatial structures. The 3 × 3 convolution filter in the expand layer identifies the spatial representation. The model becomes more descriptive by integrating these two distinct size filters while running on lower parameters. Hence, fire modules reduce the computational load by reducing the parameter maps and building a smaller CNN network that can preserve a higher degree of accuracy. The total parameters in our proposed method are one million parameters, which are 3, 5, 4.5, 3, and 28 times smaller than SegNet, U-net, U-SegNet, U-net++, and CE-net networks, respectively. The training time for the proposed method for the OASIS dataset was 50% of that of the U-net++ and CE-net methods. Compared to traditional approaches, a reduction in memory requirements would result in a substantial decrease in energy and processing requirements.

### Ablation Study

We conducted an ablation study on the three simplified versions of the proposed modules to investigate the influence of each selection on the segmentation performance as follows: (i) Squeeze U-SegNet, (ii) Squeeze U-SegNet with multi-scale input, (iii) Squeeze U-SegNet with multi-global attention and (iv) multi-scale Squeeze U-SegNet with multi-global attention (proposed method). The Squeeze U-SegNet was obtained by replacing each convolution block by fire module in the conventional U-SegNet. The second network proposes that the encoder of the Squeeze U-SegNet includes a multi-scale input layer. This is achieved by max-pooling the input and performing parallel convolution with 1 × 1, 3 × 3 kernels, and concatenating these multi-scale features. These fused multi-scale features are concatenated with the corresponding fire module output and fed as input for further max-pooling operations. This process is repeated for all encoding layers. The multi-scale feature module extracts neighbor scale information of global features more precisely while filtering out irrelevant information. The impact of the attention mechanism is explored in the third network, where GAMs are integrated at both the encoder and decoder, forming a multi-attention network. Finally, the multi-scale squeeze U-SegNet with multi-global attention referred to as the proposed method incorporates semantic guidance by combining all the proposed modules. Table 4 lists the results of the individual contributions of different components to segmentation performance. The fire module-based model shows a large decrease in the requirement of learnable parameters with reduced computation time for model training while maintaining network accuracy. We observe that, compared to the baseline squeeze U-SegNet, the performance of the models integrated with the multi-scale feature fusion input scheme and with the multi-attention modules is improved by 1.5% and 2%, respectively. Although the multi-scale feature fusion shows a slight increase in the DSC, its contribution combined with GAM provides more network efficiency. Furthermore, the combination of both multi-scale and multiple global attention strategies boosts the performance and yields the best values in the three metrics: 96% (DSC), 91% (JI), 3.1(HD), and with the lowest MSE of 0.003. These results represent an improvement of 2% in DSC compared to the baseline U-SegNet [20], showing the efficiency of the proposed multi-scale guided multi-GAM compared to individual components.

We also investigated the effects of patch size in terms of training time and segmentation performance. The experiments were performed on the OASIS dataset for three distinct patch sizes (128 × 128, 64 × 64, and 32 × 32). Table 5 lists the output of the segmentation in terms of the DSC with respect to different patch sizes. It can be observed that smaller patches result in better performance. This is because smaller patches create more training data for the network to train. Moreover, local regions can be restored more accurately. Furthermore, when the patch size is 128 × 128, it takes 1.1 h to train the model, whereas the training time doubles for 32 × 32 patches with almost identical accuracy. We, therefore, concluded that a patch size of 128 × 128 provides a fair tradeoff between the DSC score and the computational time needed to train the model, based on the results in Table 5.

## 5. Conclusions and Future Works

This paper proposes multi-scale feature extraction with novel global attention-based learning based on the U-SegNet architecture for brain MRI segmentation. The multi-scale data provide rich spatial information and improve the robustness of feature extraction. The global attention module provides the global context as guidance for low-level features to select category localization details. Squeeze and expand layers lead to the generation of one million parameters, which are 3, 5, 4.5, 3, and 28 times smaller than SegNet, U-net, U-SegNet, U-net++, and CE-net networks, respectively. Our proposed network obtains the best DSC value of 96%. The training time for the proposed method for the OASIS dataset is 50% of that of the U-net++ and CE-net methods. Our validation proves that the network operating on patch-wise input, integrated with multi-scale global attention and fire modules, will yield an efficient brain MRI segmentation model. The proposed model can be easily extended to complex network architectures owing to flexibility and adaptability with faster computation. Hence, a three-dimensional (3D) segmentation model can be devised using the extended model of the proposed architecture as future works.

## Figures and Tables

**Table 1:** Summary of OASIS and IBSR datasets used in our experiments.

Dataset	No. of Subjects			Experiment Data	
	Male	Female	Total	Training Set	Testing Set
OASIS	160	256	416	120	30
IBSR	14	4	18	12	6

**Table 2:** The formulation of evaluation metrics.

Dice similarity coefficient (*DSC*)	D(X,Y) = 2$\frac{\left|X\right|\cap \left|Y\right|}{\left|X\right|+\left|Y\right|}$
Jaccard Index (*JI*)	JI(X,Y) = $\frac{DSC}{2-DSC}$
Hausdorff distance (*HD*)	$HD=max\times \left\{\begin{array}{c} \underset{x\in X}{max}\;\underset{y\in Y}{min}\;D\left\{X,Y\right\},\\ \underset{y\in Y}{max}\;\underset{x\in X}{min}\;D\left\{X,Y\right\} \end{array}\right\}$
Mean squared error (*MSE*)	*MSE* = $\frac{1}{RC}$ $\sum_{i=1}^{R}\sum_{j=1}^{C}{\left(X-Y\right)}^{2}$

**Table 3:** Comparisons between the segmentation accuracy for the proposed method and conventional methods on OASIS and IBSR datasets.

OASIS									
Axial Plane									
Method	WM			GM			CSF		
	DSC	JI	HD	DSC	JI	HD	DSC	JI	HD
**SegNet [20]**	0.89 ± 0.087	0.83 ± 0.096	4.74 ± 0.077	0.86 ± 0.069	0.80 ± 0.089	4.69 ± 0.053	0.85 ± 0.048	0.80 ± 0.068	4.12 ± 0.079
**U-net [4]**	0.93 ± 0.059	0.89 ± 0.068	4.16 ± 0.064	0.92 ± 0.048	0.87 ± 0.038	4.24 ± 0.046	0.901 ± 0.076	0.85 ± 0.055	3.82 ± 0.039
**U-SegNet [21]**	0.94 ± 0.048	0.90 ± 0.055	3.91 ± 0.057	0.93 ± 0.056	0.88 ± 0.058	4.11 ± 0.033	0.92 ± 0.024	0.86 ± 0.093	3.64 ± 0.047
**U-net++ [23]**	0.95 ± 0.075	0.91 ± 0.042	3.78 ± 0.048	0.94 ± 0.035	0.89 ± 0.072	3.84 ± 0.025	0.93 ± 0.039	0.87 ± 0.046	3.56 ± 0.036
**CE-net [29]**	0.95 ± 0.039	0.91 ± 0.074	3.65 ± 0.050	0.94 ± 0.042	0.90 ± 0.041	3.57 ± 0.044	0.93 ± 0.043	0.88 ± 0.037	3.21 ± 0.061
**Proposed**	0.97 ± 0.025	0.92 ± 0.062	3.13 ± 0.037	0.95 ± 0.029	0.91 ± 0.033	3.16 ± 0.030	0.94 ± 0.022	0.90 ± 0.043	2.44 ± 0.038
**Coronal Plane**									
**SegNet [20]**	0.87 ± 0.098	0.83 ± 0.038	5.21 ± 0.023	0.85 ± 0.044	0.80 ± 0.068	5.49 ± 0.053	0.83 ± 0.079	0.79 ± 0.056	5.87 ± 0.084
**U-net [4]**	0.94 ± 0.065	0.89 ± 0.049	4.88 ± 0.042	0.93 ± 0.057	0.88 ± 0.056	4.95 ± 0.042	0.92 ± 0.063	0.86 ± 0.029	5.34 ± 0.073
**U-SegNet [21]**	0.95 ± 0.049	0.90 ± 0.029	4.23 ± 0.039	0.94 ± 0.081	0.88 ± 0.043	4.48 ± 0.088	0.92 ± 0.054	0.87 ± 0.047	4.97 ± 0.039
**U-net++ [23]**	0.94 ± 0.076	0.89 ± 0.073	4.05 ± 0.047	0.93 ± 0.042	0.87 ± 0.037	4.29 ± 0.044	0.93 ± 0.048	0.88 ± 0.036	4.72 ± 0.043
**CE-net [29]**	0.95 ± 0.031	0.90 ± 0.026	3.98 ± 0.076	0.94 ± 0.038	0.89 ± 0.040	4.17 ± 0.071	0.93 ± 0.039	0.89 ± 0.029	4.17 ± 0.050
**Proposed**	0.96 ± 0.043	0.91 ± 0.041	3.52 ± 0.024	0.96 ± 0.020	0.91 ± 0.031	3.76 ± 0.029	0.94 ± 0.015	0.90 ± 0.016	3.95 ± 0.033
**Sagittal Plane**									
**SegNet [20]**	0.88 ± 0.096	0.84 ± 0.054	5.53 ± 0.027	0.85 ± 0.083	0.81 ± 0.055	5.26 ± 0.033	0.84 ± 0.040	0.80 ± 0.077	5.69 ± 0.088
**U-net [4]**	0.94 ± 0.068	0.89 ± 0.070	5.11 ± 0.030	0.92 ± 0.074	0.89 ± 0.030	5.11 ± 0.026	0.93 ± 0.058	0.88 ± 0.053	5.21 ± 0.079
**U-SegNet [21]**	0.95 ± 0.077	0.91 ± 0.049	4.72 ± 0.042	0.93 ± 0.066	0.89 ± 0.043	4.67 ± 0.042	0.93 ± 0.037	0.89 ± 0.060	4.75 ± 0.063
**U-net++ [23]**	0.95 ± 0.060	0.90 ± 0.036	4.46 ± 0.031	0.94 ± 0.038	0.88 ± 0.029	4.32 ± 0.019	0.94 ± 0.063	0.89 ± 0.041	4.56 ± 0.041
**CE-net [29]**	0.95 ± 0.043	0.91 ± 0.064	4.13 ± 0.020	0.94 ± 0.025	0.89 ± 0.035	4.25 ± 0.034	0.94 ± 0.051	0.90 ± 0.062	4.28 ± 0.055
**Proposed**	0.96 ± 0.027	0.92 ± 0.044	3.58 ± 0.023	0.95 ± 0.011	0.91 ± 0.040	3.98 ± 0.023	0.95 ± 0.031	0.91 ± 0.018	3.43 ± 0.031
**IBSR**									
**Axial Plane**									
**SegNet [20]**	0.72 ± 0.036	0.65 ± 0.042	6.51 ± 0.65	0.75 ± 0.049	0.67 ± 0.058	6.53 ± 0.91	0.68 ± 0.099	0.59 ± 0.095	6.96 ± 0.46
**U-net [4]**	0.89 ± 0.022	0.81 ± 0.034	5.14 ± 0.51	0.91 ± 0.017	0.85 ± 0.023	4.87 ± 0.51	0.84 ± 0.065	0.75 ± 0.079	5.24 ± 0.31
**U-SegNet [21]**	0.90 ± 0.043	0.82 ± 0.051	4.76 ± 0.39	0.92 ± 0.053	0.86 ± 0.028	4.45 ± 0.65	0.84 ± 0.029	0.75 ± 0.048	4.84 ± 0.18
**U-net++ [23]**	0.88 ± 0.085	0.80 ± 0.096	5.37 ± 0.36	0.89 ± 0.037	0.83 ± 0.049	5.17 ± 0.29	0.83 ± 0.058	0.74 ± 0.082	5.34 ± 0.64
**CE-net [29]**	0.89 ± 0.055	0.81 ± 0.073	4.98 ± 0.84	0.90 ± 0.068	0.84 ± 0.083	4.95 ± 0.38	0.82 ± 0.037	0.74 ± 0.031	4.74 ± 0.93
**Proposed**	0.91 ± 0.085	0.83 ± 0.064	4.45 ± 0.57	0.93 ± 0.076	0.87 ± 0.016	4.23 ± 0.92	0.85 ± 0.097	0.77 ± 0.023	4.26 ± 0.79
**Coronal Plane**									
**SegNet [20]**	0.70 ± 0.061	0.62 ± 0.051	6.32 ± 0.82	0.73 ± 0.037	0.65 ± 0.062	6.21 ± 0.84	0.66 ± 0.054	0.57 ± 0.086	6.84 ± 0.75
**U-net [4]**	0.88 ± 0.035	0.79 ± 0.034	5.45 ± 0.67	0.90 ± 0.014	0.83 ± 0.056	5.17 ± 0.38	0.83 ± 0.012	0.76 ± 0.043	5.54 ± 0.47
**U-SegNet [21]**	0.89 ± 0.076	0.80 ± 0.046	4.61 ± 0.21	0.91 ± 0.035	0.84 ± 0.043	4.56 ± 0.19	0.84 ± 0.085	0.76 ± 0.093	4.83 ± 0.25
**U-net++ [23]**	0.88 ± 0.021	0.79 ± 0.073	5.21 ± 0.39	0.91 ± 0.093	0.85 ± 0.074	5.24 ± 0.24	0.82 ± 0.034	0.73 ± 0.067	5.73 ± 0.39
**CE-net [29]**	0.89 ± 0.034	0.80 ± 0.851	4.89 ± 0.21	0.90 ± 0.049	0.85 ± 0.068	5.98 ± 0.93	0.83 ± 0.056	0.74 ± 0.042	5.21 ± 0.20
**Proposed**	0.90 ± 0.039	0.85 ± 0.088	4.24 ± 0.43	0.92 ± 0.019	0.86 ± 0.035	4.31 ± 0.67	0.84 ± 0.078	0.76 ± 0.097	4.55 ± 0.12
**Sagittal Plane**									
**SegNet [20]**	0.71 ± 0.043	0.63 ± 0.039	6.49 ± 0.61	0.74 ± 0.073	0.66 ± 0.059	6.36 ± 0.76	0.65 ± 0.083	0.54 ± 0.092	6.99 ± 0.41
**U-net [4]**	0.86 ±0.029	0.78 ± 0.062	5.75 ± 0.37	0.89 ± 0.036	0.81 ± 0.041	5.77 ± 0.21	0.80 ± 0.071	0.73 ± 0.019	5.83 ± 0.15
**U-SegNet [21]**	0.87 ± 0.016	0.80 ± 0.048	4.89 ± 0.14	0.90 ± 0.069	0.82 ± 0.046	5.42 ± 0.06	0.81 ± 0.096	0.74 ± 0.073	4.98 ± 0.09
**U-net++ [23]**	0.85 ± 0.083	0.79 ± 0.039	4.57 ± 0.54	0.88 ± 0.077	0.79 ± 0.081	4.96 ± 0.22	0.79 ± 0.049	0.72 ± 0.069	5.60 ± 0.44
**CE-net [29]**	0.86 ± 0.054	0.80 ± 0.025	5.34 ± 0.66	0.89 ± 0.051	0.80 ± 0.037	5.86 ± 0.55	0.79 ± 0.033	0.73 ± 0.022	5.25 ± 0.37
**Proposed**	0.88 ± 0.035	0.81 ± 0.073	4.63 ± 0.36	0.91 ± 0.028	0.83 ± 0.083	5.30 ± 0.18	0.82 ± 0.053	0.75 ± 0.011	4.12 ± 0.66
**Mean Square Error (MSE)**									
	**SegNet [20]**	**U-net [4]**	**U-SegNet [21]**		**U-net++ [23]**	**CE-net [29]**		**Proposed method**	
**OASIS**	0.021	0.006	0.005		0.004	0.004		0.003	
**IBSR**	0.013	0.008	0.007		0.005	0.005		0.004	

**Table 4:** Detailed data on the number of learnable parameters and computation time for the proposed method and its three simplified versions.

Model	GM			WM			CSF				Computation Time (10 Epochs)	#Learnableparameters
	DSC	JI	HD	DSC	JI	HD	DSC	JI	HD	MSE		
**Squeeze U-SegNet**	92.05	88.06	3.52	93.37	90.42	2.8	91.65	88.06	2.0	0.006	1 h	768,788
**Squeeze U-SegNet with multi-scale input**	93.44	89.47	4.81	94.78	91.90	4.1	93.32	90.25	3.0	0.005	1.04 h	860,180
**Squeeze U-SegNet with multi global attention**	94.32	89.25	5.10	95.12	91.40	4.2	94.24	89.22	3.3	0.004	1.15 h	942,164
**Proposed method**	95.54	91.09	3.21	96.56	92.05	3.1	94.86	90.29	3.0	0.003	1.12 h	1,030,420

**Table 5:** Segmentation accuracy (%) and training time (hours) for the proposed method with different sizes of input patches.

Model	DSC			JI			Training Time (h)
	WM	GM	CSF	WM	GM	CSF	
Patch size: 128 × 128	96.56	95.54	95.73	92.05	91.09	90.23	1.1
Patch size: 64 × 64	96.74	96.13	95.49	92.49	91.58	90.54	6.7
Patch size: 32 × 32	96.91	96.85	95.73	91.88	91.76	90.71	12.4

## Data Availability

The data presented in this study are publicly available.
